# CD19^+^CD24^hi^CD38^hi^ B Cells Are Expanded in Juvenile Dermatomyositis and Exhibit a Pro-Inflammatory Phenotype After Activation Through Toll-Like Receptor 7 and Interferon-α

**DOI:** 10.3389/fimmu.2018.01372

**Published:** 2018-06-22

**Authors:** Christopher J. M. Piper, Meredyth G. Ll. Wilkinson, Claire T. Deakin, Georg W. Otto, Stefanie Dowle, Chantal L. Duurland, Stuart Adams, Emiliano Marasco, Elizabeth C. Rosser, Anna Radziszewska, Rita Carsetti, Yiannis Ioannou, Philip L. Beales, Daniel Kelberman, David A. Isenberg, Claudia Mauri, Kiran Nistala, Lucy R. Wedderburn

**Affiliations:** ^1^Centre for Rheumatology, University College London, London, United Kingdom; ^2^Centre for Adolescent Rheumatology, Arthritis Research UK, University College London Hospital and Great Ormond Street Hospital, London, United Kingdom; ^3^Infection, Inflammation and Rheumatology Section, University College London, Great Ormond Street Institute of Child Health, London, United Kingdom; ^4^NIHR Great Ormond Street Hospital Biomedical Research Centre, London, United Kingdom; ^5^Experimental and Personalised Medicine, Genetics and Genomic Medicine, University College London, Great Ormond Street Institute of Child Health, London, United Kingdom; ^6^Haematology, Specialist Integrated Haematological Malignancy Diagnostic Service (SIHMDS), Great Ormond Street Hospital for Children NHS Foundation Trust, London, United Kingdom; ^7^B Cell Physiopathology Unit, Immunology Research Area, Ospedale Pediatrico Bambino Gesù IRCSS, Rome, Italy

**Keywords:** immature transitional B cells, B cells, juvenile dermatomyositis, toll-like receptor 7, interferon alpha, interleukin-10

## Abstract

Juvenile dermatomyositis (JDM) is a rare form of childhood autoimmune myositis that presents with proximal muscle weakness and skin rash. B cells are strongly implicated in the pathogenesis of the disease, but the underlying mechanisms are unknown. Therefore, the main objective of our study was to investigate mechanisms driving B cell lymphocytosis and define pathological features of B cells in JDM patients. Patients were recruited through the UK JDM Cohort and Biomarker study. Peripheral blood B cell subpopulations were immunophenotyped by flow cytometry. The results identified that immature transitional B cells were significantly expanded in active JDM, actively dividing, and correlated positively with disease activity. Protein and RNAseq analysis revealed high interferon alpha (IFNα) and TLR7-pathway signatures pre-treatment. Stimulation of B cells through TLR7/8 promoted both IL-10 and IL-6 production in controls but failed to induce IL-10 in JDM patient cells. Interrogation of the CD40–CD40L pathway (known to induce B cell IL-10 and IL-6) revealed similar expression of IL-10 and IL-6 in B cells cultured with CD40L from both JDM patients and controls. In conclusion, JDM patients with active disease have a significantly expanded immature transitional B cell population which correlated with the type I IFN signature. Activation through TLR7 and IFNα may drive the expansion of immature transitional B cells in JDM and skew the cells toward a pro-inflammatory phenotype.

## Introduction

Juvenile dermatomyositis (JDM), a systemic autoimmune disease, is the most common idiopathic inflammatory myopathy in childhood. Symptoms include proximal muscle weakness and skin rash around the eyes (heliotrope), which are pathognomonic for JDM (1). Other organ involvement in JDM is common, with a proportion of patients developing severe complications such as interstitial lung disease, gut involvement, and calcinosis (2). Disease pathology is thought to involve autoimmune tissue attack and complement-mediated vasculopathy (3). Inflammatory infiltrate typically seen in affected muscle is composed of B cells, CD4^+^ and CD8^+^ T cells, macrophages, and dendritic cells (DCs) (4, 5). DCs are not normally present in healthy muscle tissue; however, at the onset of myositis, patients’ muscle biopsies have an abundance of CD83^+^ plasmacytoid DCs (pDC) (6, 7), which are a major source of interferon alpha (IFNα).

Several reports document a significant IFNα gene signature in peripheral blood (PB) samples from adult dermatomyositis (DM) patients (8, 9). IFNα-inducible chemokines, CXCL10 and CXCL11 levels in serum, associate strongly with mononuclear cell infiltration in JDM muscle (10) and with disease activity (8, 9, 11). As well as increasing MHC class I expression (12, 13), and facilitating antigen presentation, IFNα enhances autoantibody production and proliferation of B cells (14–16). Importantly, B cells are thought to play a role in JDM pathology through the production of myositis-specific autoantibodies (MSAs). Although it is currently unknown how autoantibodies contribute to disease pathology, specific MSAs are associated with distinct clinical phenotypes, and the frequency and types of MSAs vary between adult and juvenile forms of DM (17). The type of MSA associates with particular muscle biopsy pathology (18, 19), which is predictive of disease progression and outcome (20). To date, there is no information regarding whether IFNα affects abnormal B cell responses in JDM.

In addition to having pathogenic roles in autoimmune disease or protective roles in controlling infection, B cells have been shown to have suppressive function, through the production of IL-10 (21–23). Several IL-10-producing regulatory B cells (Bregs) populations have been identified in humans (24). These Breg populations include CD19^+^CD24^hi^CD38^hi^CD27^−^ (immature transitional B cells) (25), CD27^+^CD24^hi^ (B10) (26), CD73^−^CD25^+^CD71^+^ (B_R_1) (27), and CD27^int^CD38^+^ plasmablasts (28). Bregs are known to be diminished and/or functionally impaired in various autoimmune diseases, including systemic lupus erythematosus (SLE), systemic sclerosis, rheumatoid arthritis, and ANCA-associated vasculitis (25, 29–31). Importantly, IFNα is known to induce both the differentiation of Breg and plasmablasts in a concentration-dependent manner (32). This suggests that the type 1 interferonopathy observed in juvenile myositis could influence both regulatory and pathogenic B cell responses. The effects of type I interferon on B cell function in JDM have not been studied.

In this study, we carry out the first in-depth phenotyping of the B cell compartment in active JDM, before treatment. We demonstrate that CD19^+^CD24^hi^CD38^hi^ immature transitional B cells are expanded in JDM patients before treatment and correlate with disease severity. We show that the previously reported interferon signature in JDM is highly upregulated in B cells and positively correlates with this expansion in immature transitional B cells. Finally, we demonstrate that after activation of B cells with TLR7 agonist R848 and IFNα, the ability of JDM B cells to make IL-10 is inhibited. However, B cells from patients are able to produce IL-10 in response to alternative pathways, including activation through CD40.

## Materials and Methods

### Patient and Controls

68 patients who met the Bohan and Peter criteria for probable or definite JDM were included in this study, with a median age of 9.9 years at time of sample (Table 1), recruited through the UK JDM Cohort and Biomarker Study (JDCBS) (33–35). Inclusion in the JDCBS is offered to all patients with JDM seen in the 17 contributing centers. 99% of families consent to being in this observational study, which now has 580 cases; 6 have declined in >15 years. The 68 patient cases used in this particular study were consecutive cases available for analysis that were representative of the whole cohort. In addition, 23 age-matched child healthy controls (CHCs) were recruited. Parents and patients gave written informed consent or age-appropriate assent. The study obtained ethical approval through London-Bloomsbury and North-East Yorkshire Research Ethics Committees (MREC—01/3/022). CHC samples were donated under the North Harrow ethics committee approval (REC 11/0101). All consent was obtained in accordance with the Declaration of Helsinki. Clinical data were collected at serial data points as described (Table 2), including medications, Physicians Global Assessment (PGA; range 0–10; low scores indicate minimal disease) (36, 37), manual muscle testing of a subset of eight muscles (MMT8; range 0–80; high scores indicate no muscle weakness) (38), Childhood Myositis Assessment Scale (CMAS; range 0–52; high scores indicate no weakness) (39), and serum creatine kinase levels (U/l) (Table 3). Blood was collected before, and at serial time points during treatment, as indicated. Patient and CHC demographics are summarized in Table 1. Patients were stratified into pre-treatment, <6, 7–30, and >30 months on-treatment. All patients were treated with steroids and methotrexate at the start of therapy.

**Table 1:** Demographic of the juvenile dermatomyositis (JDM) cohort and child healthy controls (CHCs).

Patient characteristics	JDM patients	Controls	*P* Value

	All JDM samples (*n* = 111)	CHC (*n* = 26)	
Number of patients	68	26	
Age at onset (years), median [IQR]	5.61 [4.19–9.38]	N/a	N/a
Age at diagnosis (years), median [IQR]	5.53 [4.90–9.04]	N/a	N/a
Age at sample (years), median [IQR]	9.9 [8.4–12.9]	7.50 [6.33–11.00]	0.8614
Sex (F/M)	43/25	13/13	0.0835

**Table 2:** Demographic, clinical, and serological features of the JDM cohort.

Patient characteristics	JDM patients	
	Pre-treatment samples (*n* = 20)	On-treatment samples (*n* = 93)
Number of patients	20	48
Number of patients with 1 unique sample	6	22
Number of patients with 2 or more samples	14^a^	26

**Clinical features, median [IQR]**		
Physicians Global Assessment (0–10)	6.70 [5.73–7.68]	0.85 [0.20–1.63]
MMT8 (0–80)	29.00 [28.00–42.00]	80 [76–80]
CMAS (0–53)	11.5 [4.50–22.75]	51 [47–53]
Creatine kinase U/l (>0) (measured in serum at time of PBMC sample)	652.5 [265.00–3,893.25]	87 [48–128]

**Medications (at time of sample) *n*(%)**		
Azathioprine		17 (18.27)
Cyclophosphamide		17 (18.27)
Oral prednisolone		60 (64.52)
Methotrexate		59 (63.44)
Intravenous prednisolone		32 (34.41)
Other drugs		11 (11.83)

*MMT8, Manual Muscle Testing of 8 groups; CMAS, Childhood Myositis Assessment Scale; PBMC, peripheral blood mononuclear cells; JDM, juvenile dermatomyositis*.

*^a^Serial samples were used from 40 patients, 14 of these patients had pre- and on-treatment samples*.

**Table 3:** Measures of disease activity.

Disease activity tool	Score range	Inactive disease cutoff
Creatine kinase	>0	≤150 U/l
Physicians Global Assessment	0–10	≤0.2
Childhood Myositis Assessment Scale	0–53	≥48
Manual Muscle Testing of 8 groups	0–80	≥78

### Cell Isolation and Culture

Peripheral blood mononuclear cells (PBMC) were isolated by density centrifugation using Ficoll-Paque Plus (GE Healthcare). PBMC were stored in 90% FCS (BioTech) and 10% DMSO (Sigma) and cryopreserved in liquid nitrogen until use. Cells were cultured with RPMI 1640 (Sigma) containing l-glutamine and NaHCO_3_, supplemented with 10% FCS and 1% penicillin/streptomycin (Sigma).

### Flow Cytometry and Cell Sorting

Flow cytometry was performed with the following directly conjugated antibodies: CD10 (HI10a, BioLegend), CD19 BV785 (HIB19; BioLegend), CD24 APC (SN3 A5-2H10; Thermo Fisher Scientific), CD27 PECy7 (O323; Thermo Fisher Scientific), CD38 BV605 (HIT2; BioLegend), IgD FITC (IA6-2; Thermo Fisher Scientific), and IgM Pacific Blue (SA-DA4; Thermo Fisher Scientific). LIVE/DEAD fixable blue/yellow Dead Cell Stain (Thermo Fisher Scientific) was used to exclude dead cells from flow cytometric analysis. For measurement of *ex vivo* intra-nuclear Ki-67 (B56; BD Pharmingen), cells were fixed for 20 min with FOXP3 Fixation buffer (Thermo Fisher Scientific), and Ki-67 was added in permeabilization buffer. B cell subsets were sorted using a cell sorter (FACSAria; BD Pharmingen) by using CD19 BV785, CD24 APC, CD27 PECy7, and CD38 BV605, as above. Dead cells were excluded by the use of 4,6-diamidino-2-phenylindole (DAPI; Sigma). Sort purity of B cells was routinely >95%. For detection of TLR7 and cytokines, intracellular fixation/permeabilization kit (Thermo Fisher Scientific) was used. PBMC were stained for TRL7 (533707; BioTechne) or a monoclonal mouse IgG2a PE isotype control (BioTechne) for 40 min in permeabilization buffer.

For detection of intracellular IL-6 (MQ2-13A5; Thermo Fisher Scientific) and IL-10 (JES3-19F1; BD Pharmingen), PBMC/B cells were cultured with CD40L transfected Chinese Hamster Ovary (CHO) cells for 72 h as previously described (25), or for 48 h with R848 (TLR7/8 agonist) at 1 µg/ml (Invivogen) ± recombinant IFNα at 1,000 IU/ml (PBL assay Science). During the last 4 h of culture, cells were incubated in the presence of PMA (50 ng/ml), Ionomycin (250 ng/ml), and Brefeldin A (5 μg/ml) (Sigma). Flow cytometric data were collected on an LSRII or LSR Fortessa (BD Pharmingen) using FACS Diva software. Data were analyzed using Flowjo (Tree Star).

### Analysis of Kappa-Deleting Recombination Excision Circle (KREC) Content

Immature transitional, mature, and memory B cells were sorted and DNA was extracted using a QIAamp Blood DNA Mini Kit (Qiagen), according to the manufacturer’s instructions. Quantitative real time PCR (qPCR) was carried out on the DNA samples as described (40), with a standard curve method of analysis, using serial dilutions of a known quantity (10^6^, 10^5^, 10^4^, 10^3^, 10^2^, and 10 copies) of a linearized plasmid containing segments of T cell receptor alpha constant (TRAC), KRECs, and T cell receptor excision circles. Details of the plasmid and primer/probe sequences used were as described previously (41).

The quantity of KRECs per 10^6^ cells was calculated by the following equation, whereby *n* is the total amount of KRECs per 10^6^ cells; *k* is the mean quantity of KRECs, and *t* is the mean quantity of TRAC

$$n=\frac{k}{t\text{​}/\text{​}2}\times 10^{6}$$

### Measurement of Cytokine/Chemokine Concentrations

Supernatants were taken from cell cultures before the addition of PMA, Ionomycin, and Brefeldin A. IL-6 and IL-10 cytokine concentrations were measured using a human IL-6 DuoSet ELISA kit (R&D Systems) and Ready-Set-Go human IL-10 ELISA kit (eBioscience) according to the manufacturer’s instructions. Sera from patients were tested for CXCL10, CXCL11, MCP-1, and MCP-2 *via* Luminex multiplex cytokine array (42).

### RNA Sequencing

Patient and control CD19^+^ cells were sorted by flow cytometry (FACS Aria III). DAPI was used to exclude dead cells. Sorted B cell RNA was extracted using the Arcturus PicoPure RNA Isolation Kit (Thermo Fisher Scientific). Library preparation and sequencing were performed at UCL genomics, and data were analyzed using a customized pipeline (see Supplementary Methods in Supplementary Material for full methodology). RNAseq data are available from ArrayExpress, accession number E-MTAB-5616.

### Statistical Analysis

Data, excluding RNAseq, were analyzed using GraphPad Prism 6. Expression analysis was carried out using R version 3.2.2, and differential gene expression was analyzed using edgeR (43, 44). One-way or two-way analysis of variance (ANOVA) was used to assess significance of differences between group means (≥3 groups), and unpaired Student’s *t*-test was used to assess significance between two groups. Bonferroni corrections were applied for multiple comparisons using ANOVA, or Sidak multiple comparisons test was applied for two-way ANOVA. Pearson’s/Spearman’s correlation coefficients were used to assess correlations. Bar graph data represented as mean ± SEM. For all figures, *p* values are represented as follows: *p < 0.05; **p < 0.01; and ***p < 0.001.

## Results

### Immature Transitional B Cells Are Expanded in JDM Patients Before Treatment

**Figure 1:**
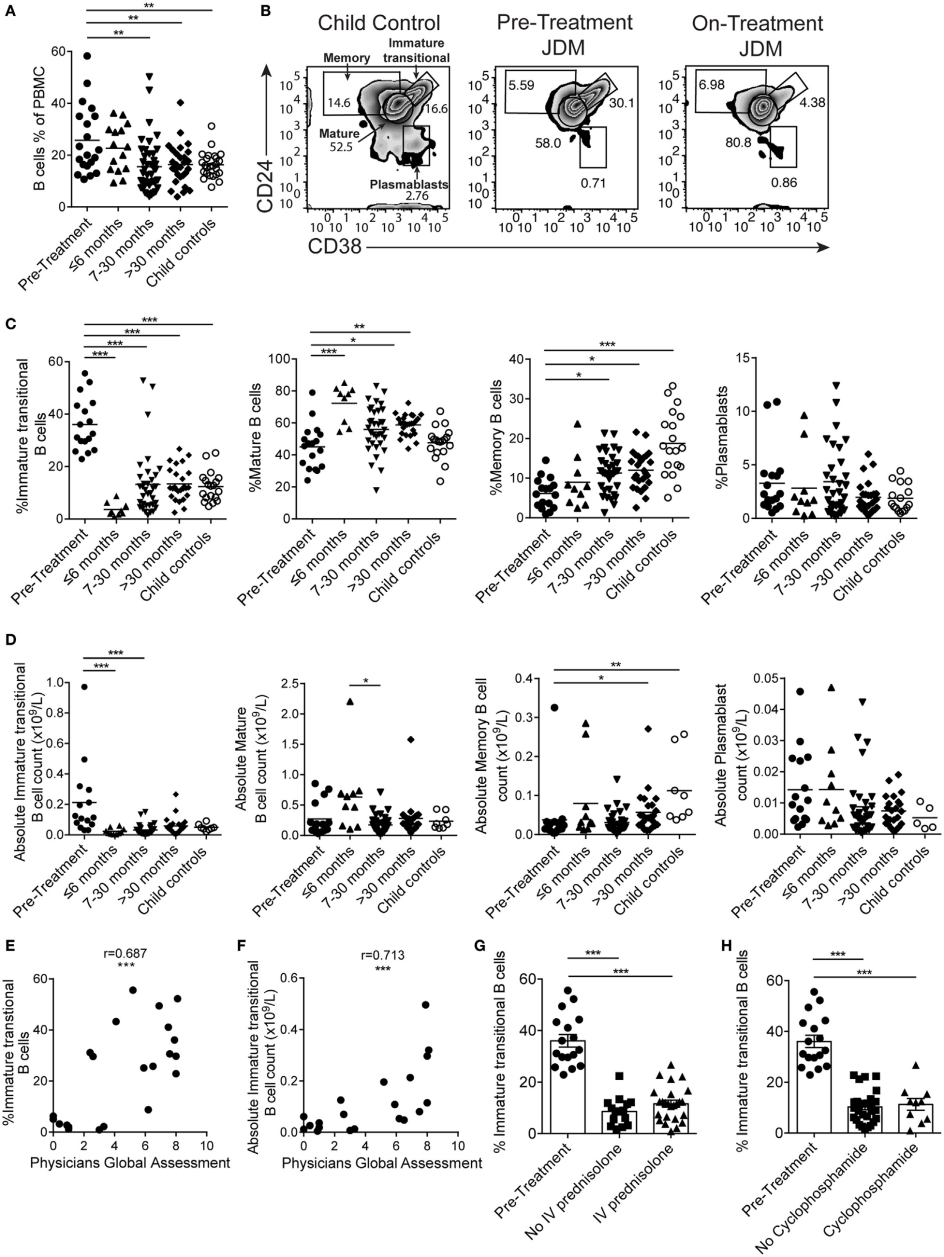
Immature transitional B cells are significantly expanded in juvenile dermatomyositis (JDM) patients before treatment. Peripheral blood mononuclear cells (PBMC) from patients and age-matched controls were analyzed by flow cytometry and B cell data compared across disease course and against clinical measures. **(A)** B cell (CD19^+^ cells within all PBMC) frequencies summarized according to time since treatment start in patients compared with controls. **(B)** Representative flow cytometry plots of B cell subsets, identified using expression of CD24 and CD38, shown for child controls (left plot), JDM pre-treatment (middle plot), and JDM (<6 months) on-treatment (right plot). Bar graphs of **(C)** frequency and **(D)** absolute numbers of immature transitional, mature, and memory B cells and plasmablasts (left–right) in patients and controls. For patients up to 6 months into treatment, **(E)** frequency and **(F)** absolute numbers of immature transitional B cells were correlated to Physicians Global Assessment. **(G)** Immature transitional frequency in JDM patients pre-treatment and after treatment with intravenous prednisolone or **(H)** cyclophosphamide. Pearson *r* values are shown for panel **(E)**, Spearman *r* values for panel **(F)**. For panels **(A,C,D)**, lines represent mean values. For panels **(G,H)**, bars represent mean ± SEM (*p < 0.05, **p < 0.01, and ***p < 0.001).

B cells were first reported to be expanded in the PB of JDM patients in 1976 (45). However, since this initial observation, detailed characterization of the B cell compartment and subpopulations in JDM, before treatment, has not been performed. In agreement with previously published data, there was a significant increase in the percentage of CD19^+^ B cells in pre-treatment JDM patients compared with age-matched CHC; a phenomenon that was normalized by 7–30 months on-treatment (Figure 1A). Notably, there was no overall lymphocytosis in these patients (Figure S1A in Supplementary Material). To characterize the B cell compartment, we evaluated the frequency and absolute number of B cell subpopulations, defined as immature transitional (CD19^+^CD24^hi^CD38^hi^), mature (CD19^+^CD24^int^CD38^int^), memory (CD19^+^CD24^hi^CD38^lo^) B cells, and plasmablasts (CD19^+^CD24^lo^CD38^lo^) (46) within total CD19^+^ B cells in pre-treatment JDM PBMC and on-treatment JDM PBMC (>6, 7–30, and <30 months), compared with CHC PBMC by flow cytometry (Figure 1B). Analysis of B cell subset frequencies revealed that there was a significant expansion in the percentage and absolute number of CD19^+^CD24^hi^CD38^hi^ immature transitional B cells, which was significantly decreased within the first 6 months of treatment. After 6 months on-treatment, equivalent levels of immature transitional B cells were seen in CHC and patients (Figures 1C,D). Importantly, the expanded CD19^+^CD24^hi^CD38^hi^ B cells observed pre-treatment also expressed high levels of IgD, IgM, CD10 and were negative for CD27 (IgD^+^IgM^+^CD10^+^CD27) confirming their immature transitional B cell phenotype (Figures S1B,C in Supplementary Material). In both CHC and JDM pre-treatment, mature and memory B cells did not express CD10 (data not shown). There was a reduction in percentage and absolute number of memory B cells in pre-treatment JDM patients compared with CHC, a defect that was also normalized by treatment. Moreover, mature B cell frequency increased after treatment. No differences in the percentage or absolute number of plasmablasts between pre-treatment JDM patients, post-treatment JDM patients, and CHC could be observed (Figures 1C,D).

In accordance with previously published results (47), immature transitional B cell frequency significantly decreased with age in CHC (*p* < 0.05); interestingly, however, there was no such correlation between immature transitional B cell frequency and age in JDM patients (Figure S1D in Supplementary Material), suggesting that the expansion of immature transitional B cells is independent of age in JDM. Notably, the frequency of mature B cell, memory B cell, and plasmablasts did not correlate with age in either pre-treatment JDM patients or CHC (Figure S1D in Supplementary Material).

Given that B cell subset distribution is abnormal in JDM patients pre-treatment, we next explored the association of these B cell subsets in relation to JDM disease severity. This analysis showed that PGA, the gold standard clinical measure of disease activity in JDM, was significantly correlated with the frequency of the immature transitional B cell subset, whether analyzed as proportion of B cells or absolute numbers *p* < 0.001 (Figures 1E,F). The strong positive correlation between the frequency of immature transitional B cells and PGA was specific to this population (Figures S1E,F in Supplementary Material) while the frequency of memory B cells correlated negatively with disease severity. To assess the effects of different treatments, we analyzed patients stratified by treatment with or without intravenous (IV) prednisolone and cyclophosphamide (12/17 patients had both treatments). All patients, whether they received IV prednisolone or cyclophosphamide, were on other treatments. The expansion of the immature transitional population seen in pre-treatment significantly decreased with treatment. There was no difference in this population when stratified with or without IV prednisolone or cyclophosphamide (Figures 1G,H). Taken collectively, these results show that B cell subset abnormalities in JDM, characterized by an expansion of immature transitional B cells, are normalized by treatment and these correlate with changes in disease activity.

To confirm that abnormalities in B cell subset distribution were normalized by treatment, we next assessed B cell subset frequency in serial samples from a set of patients (Figure S2 in Supplementary Material). In concordance with the results in Figures 1C,D, we found that there was a trend for a reduction in immature transitional B cells, while memory B cells significantly increased after treatment (*p* < 0.05). No differences were seen in mature B cells and plasmablasts (Figure S2 in Supplementary Material). These results confirm that treatment normalizes immature transitional B cell subset frequency in JDM patients and that this cannot be accounted for by the normal aging of the immune system as seen in healthy children.

### Immature Transitional B Cells Are Highly Proliferative in Pre-Treatment JDM Patients

**Figure 2:**
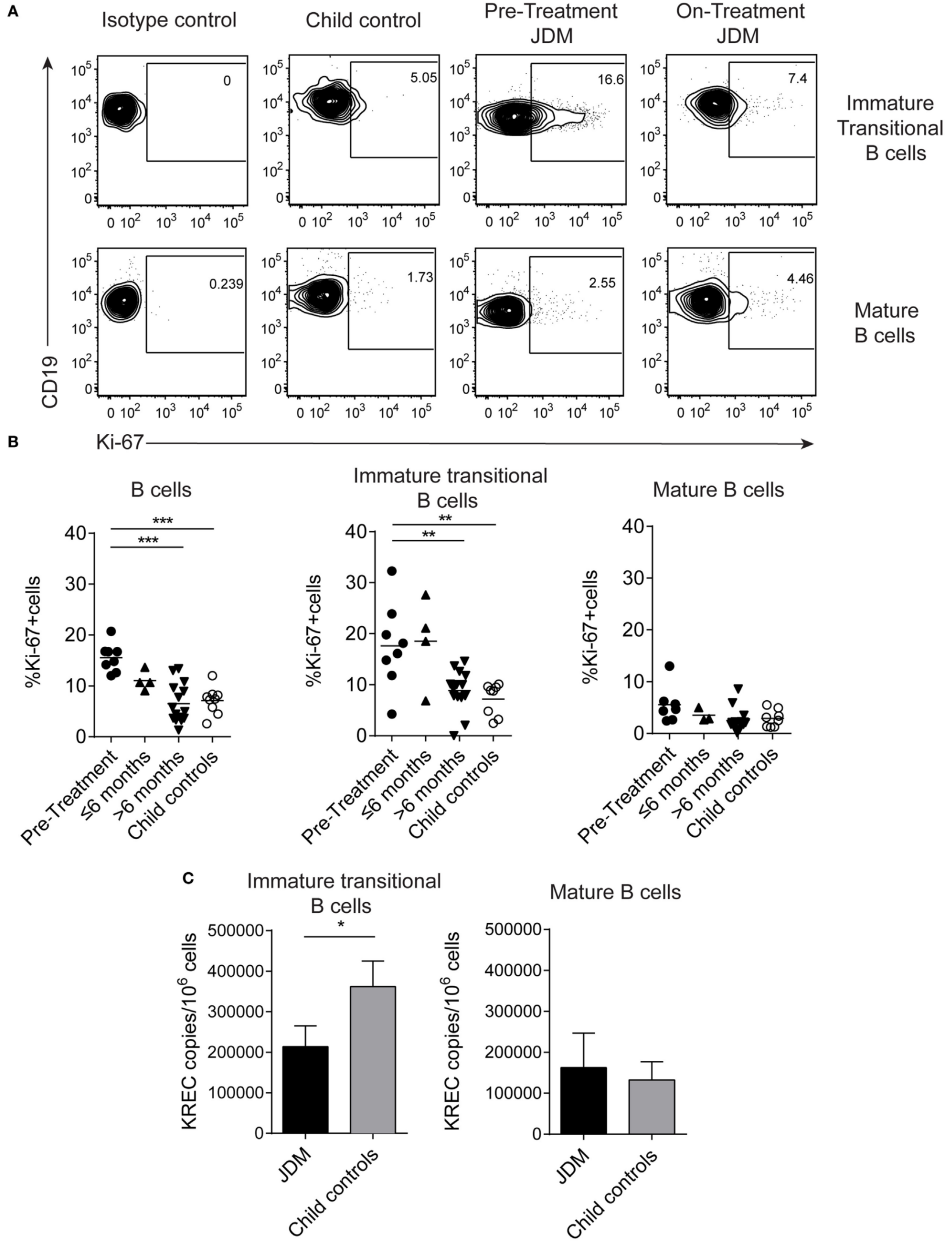
Immature transitional B cells are highly proliferative in juvenile dermatomyositis (JDM) patients before treatment. Peripheral blood mononuclear cell (PBMC) samples were stained *ex vivo* for B cell surface markers CD19, CD24, and CD38 and the intra-nuclear marker of proliferation, Ki-67. **(A)** Representative flow cytometry plots of Ki-67 expression in immature transitional (top row) and mature B cells (bottom row), in patients and controls. **(B)** The frequency of Ki-67^+^ B cells was summarized for total B cells, immature transitional B cells, and mature B cells (left–right) for patients split according to time from treatment start, and for child controls. **(C)** B cell subpopulations were sorted from patients on-treatment (*n* = 3) and child controls (*n* = 3) PBMC. DNA extracted from immature transitional (CD19^+^CD24^hi^CD38^hi^CD27^−^) and mature B cells (CD19^+^CD24^int^CD38^int^CD27^−^) were assessed for the levels of kappa-deleting recombination excision circles (KRECs). KREC copies per 10^6^ cells were calculated for immature transitional and mature B cells. Lines represent mean values for panel **(B)**. For panel **(C)**, bars represent mean ± SEM (*p < 0.05, **p < 0.01, and ***p < 0.001).

As immature transitional B cells were significantly expanded in JDM patients pre-treatment compared with CHC, we next explored whether these cells were more proliferative in JDM. For this analysis, we used two complementary approaches. First, B cells were stained for expression of Ki-67 using flow cytometry. Ki-67 identifies cells in all stages of cell division, except resting G_0_ phase and therefore identifies proliferating cells. Increased proliferation was specifically observed in immature transitional B cells in pre-treatment patients (Figures 2A,B). Second, B cell subpopulations were sorted from on-treatment JDM patients and CHC, and KREC content was measured by PCR. KRECs are generated following rearrangement of the Ig kappa chain locus of the B cell receptor. As KREC DNA is not replicated, every cycle of division leads to a halving in the daughter cell’s KREC content (48), allowing quantification of cell division from B cells, providing insight into the replication history of a cell population. Sorted immature transitional B cells from JDM patients had a significantly lower KREC content compared with CHC, signifying more cell division, while no difference was observed in mature B cells (Figure 2C). KREC content was undetectable in memory B cells and plasmablasts, due to the number of divisions these populations undergo *in vivo* (data not shown). These data suggest that immature transitional B cells from pre-treatment JDM patients proliferate more than their CHC counterparts and that this phenomenon is specific to immature transitional B cells in JDM.

### Upregulation of the Type I Interferon Signature in JDM B Cells

**Figure 3:**
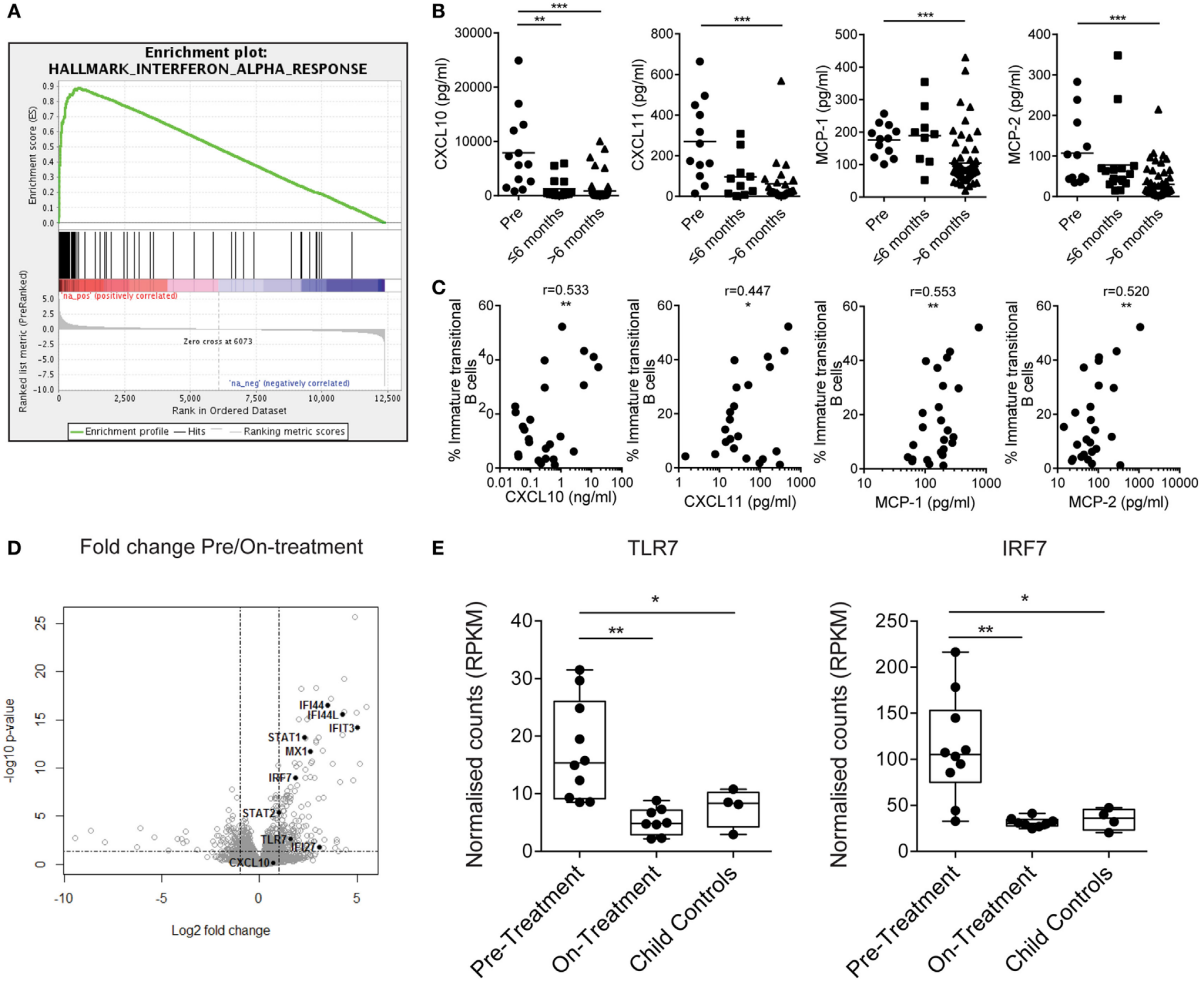
Juvenile dermatomyositis (JDM) B cells have a prominent interferon alpha (IFNα) and TLR7-pathway signature. **(A)** Gene set enrichment analysis plot showing the hallmark IFNα response gene-set in pre- vs on-treatment patient B cells. **(B)** Patient sera from pre- and on-treatment JDM patients were analyzed for chemokines known to be downstream of the IFNα pathway. Serum concentrations of CXCL10, CXCL11, MCP-1, and MCP-2 (left–right) measured by Luminex multiplex array are summarized according to time since treatment start. Patient sera and peripheral blood mononuclear cells were collected on the same day. **(C)** Immature transitional B cell frequency and serum levels of CXCL10, CXCL11, MCP-1, and MCP-2 (left–right) were correlated in JDM patients (pre-/on-treatment). **(D)** Volcano plot highlighting differentially expressed genes downstream of the IFNα pathway in pre- vs on-treatment patients. **(E)** Normalized counts (reads per kilobase of transcript per million mapped reads—RPKM) for pre-treatment, on-treatment patients and child controls are shown for TLR7 and interferon-regulatory factor 7 (IRF7). For panel **(E)**, *N* = 10 for pre-treatment, 9 for on-treatment, and 4 for child controls. For panel **(B)**, lines represent mean values. For panel **(C)**, Pearson *r* values are shown. For panel **(E)**, data are represented as box and whisker plots with means. The whiskers represent minimum and maximum data points (*p < 0.05, **p < 0.01, and ***p < 0.001).

To understand what drives immature transitional B cell expansion in JDM patients pre-treatment, we carried out RNA sequencing transcriptome analysis of B cells isolated from nine pre- and nine on-treatment patients (median time on-treatment 11 months) and four CHC. Gene set enrichment analysis of Hallmark pathways in the ranked gene lists revealed that the IFNα response was the most upregulated pathway in pre- vs on-treatment isolated B cells (Figure 3A). The normalized enrichment score was 3.03 with an FDR *q*-value of <0.0001. The 20 most significant differentially expressed genes between pre- vs on-treatment patients, pre-treatment vs CHC, and on-treatment vs CHC are summarized in Tables S1–S3 in Supplementary Material. The IFNα signature was validated at protein level using analysis of patient serum by Luminex multiplex array (49, 50). Pre-treatment patients had high serum concentrations of CXCL10, CXCL11, MCP-1, and MCP-2, which reduced in patients on-treatment for >6 months (Figure 3B). Serum levels of chemokines were also correlated with the frequency of immature transitional B cells (Figure 3C). No significant positive correlation was seen with mature or memory B cells with CXCL10, CXCL11, MCP-1, or MCP-2 (Figures S3A,B in Supplementary Material), or in plasmablasts (data not shown). However, there was a significant negative correlation between MCP-1 and mature B cells. Of note, serum and PBMC samples analyzed were from samples obtained on the same day.

IFNα regulates the expression of TLR7 and enhances the response to endogenous TLR7 ligands by B cells, and if overexpressed, can lead to the development of autoreactive B cells and autoantibody production (51, 52). Since TLR7 is linked to pathogenic B cells and the development of RNA autoantibody production in mouse models of SLE (53, 54), we hypothesized that the TLR7 pathway is important to B cell pathology in JDM. Interferon-regulatory factor 7 (IRF7) is activated both through type 1 IFN and through pattern recognition receptors, including TLR7 (55). RNAseq identified upregulation of *TLR7* and *IRF7* in pre- vs on-treatment patients (Figure 3D). Normalized counts for *TLR7* and *IRF7* were significantly higher in pre-treatment patients compared with on-treatment patients and CHC (Figure 3E). In addition, at protein level transitional immature B cells displayed a trend for increased TLR7 in active JDM patients, compared with CHC (Figures S4A,B in Supplementary Material). Taken collectively, these data show that the IFNα signature strongly associates with immature transitional B cell frequency, and TLR7 and its downstream signaling component IRF7 are upregulated in JDM B cells.

### TLR7 and IFNα Promote IL-6, but Not IL-10 Production in B Cells From JDM Patients

Since our results identify an expansion of immature transitional B cells and upregulation of the type I interferon signature in JDM B cells, we next examined the effects of IFNα and TLR7 on healthy and JDM B cells. To assess B cell response to TLR7 ligation, we cultured purified B cells for 48 h with or without IFNα and quantified B cell IL-6 and IL-10 expression. These cytokines were selected as readouts because IL-6 is known to associate with disease pathology in myositis (9, 56) and IL-10 is a marker of Bregs. It has been shown previously that culturing healthy control B cells with IFNα increases the CD19^+^CD24^hi^CD38^hi^ (Breg containing) population and promotes Breg IL-10 production. In addition, IFNα can promote the development of plasmablasts in a dose-dependent fashion (32). Stimulating B cells with R848 (TLR7/8 agonist) and IFNα showed a trend toward an increase in the percentage of CD19^+^CD24^hi^CD38^hi^ cells from CHC, but not in active on-treatment JDM patients [with PGA > 3.5 (range 0–10)] (Figures S5A,B in Supplementary Material). No differences in cell death *in vitro* were observed between patients and CHC (data not shown).

**Figure 4:**
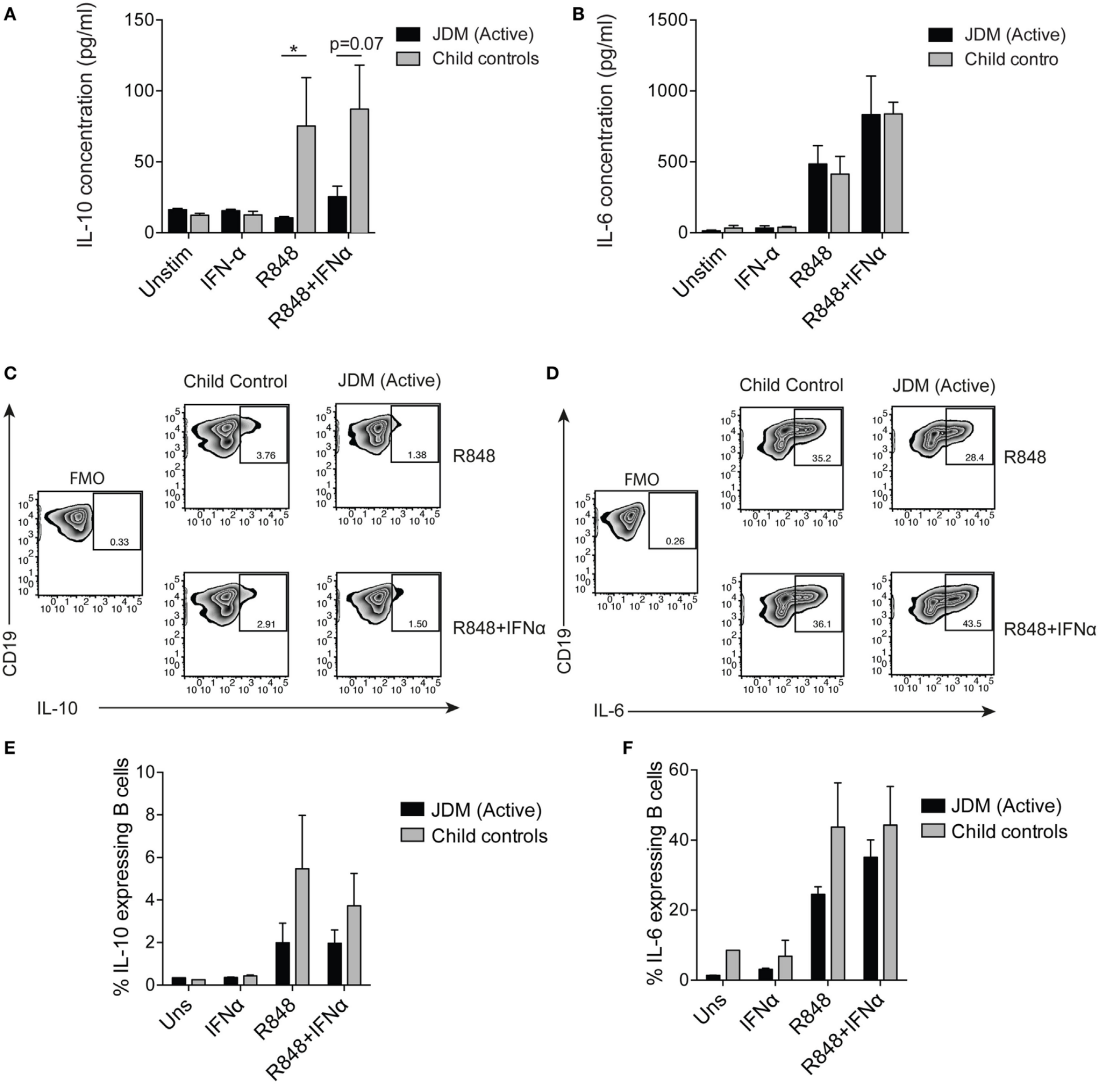
Juvenile dermatomyositis (JDM) B cells fail to induce IL-10 after TLR7 stimulation. B cells purified from active on-treatment patients [patients with flares had high disease activity (Physicians Global Assessment > 3.5) and were >6 months into treatment], and age-matched child control peripheral blood mononuclear cells, were stimulated with interferon alpha (IFNα) (1,000 IU/ml), 1 µg/ml R848 (a TLR7/8 agonist) or both, for 48 h. Culture supernatants taken from total B cells at 48 h were analyzed for **(A)** IL-10 and **(B)** IL-6 concentrations for patients and controls. Representative flow cytometry plots showing percentages of **(C)** IL-10^+^ and **(D)** IL-6^+^ B cells after 48 h stimulation with R848 (top row) and R848^+^ IFNα (bottom row). FMO plots are also shown for each cytokine. The frequencies of **(E)** IL-10^+^ and **(F)** IL-6^+^ B cells for child controls and active JDM patients are summarized. A two-way analysis of variance with Sidak multiple comparisons test was used for panels **(A,B,E,F)**. *N* = 3 for both patients and controls. For panel **(A,B,E,F)**, bars represent mean ± SEM (*p < 0.05).

To compare flow cytometry data with secreted protein, concentrations of IL-6 and IL-10 were quantified from cell culture supernatants by ELISA. TLR7 ligation induced IL-10 production in controls, but IL-10 production was significantly diminished in supernatants from patients on-treatment (Figure 4A). No difference between patients and controls was observed in secreted IL-6 (Figure 4B). Furthermore, addition of IFNα to cultures increased B cell IL-6 secretion by both patient and control B cells (Figure 4B). Analysis of IL-10 and IL-6 by intracellular staining and flow cytometry followed the same trend, with decreased IL-10 in JDM patients and no significant difference in IL-6 between patients and controls (Figures 4C–F). These data suggest IL-10 production is diminished after TLR7 activation in JDM B cells. However, IL-6 expression in JDM B cells is comparable to healthy controls, suggesting an imbalance in pro-inflammatory and anti-inflammatory cytokine responses in JDM B cells in response to TLR7 activation.

### CD40L-Stimulated JDM B Cells Have No Defect in IL-10 Production

**Figure 5:**
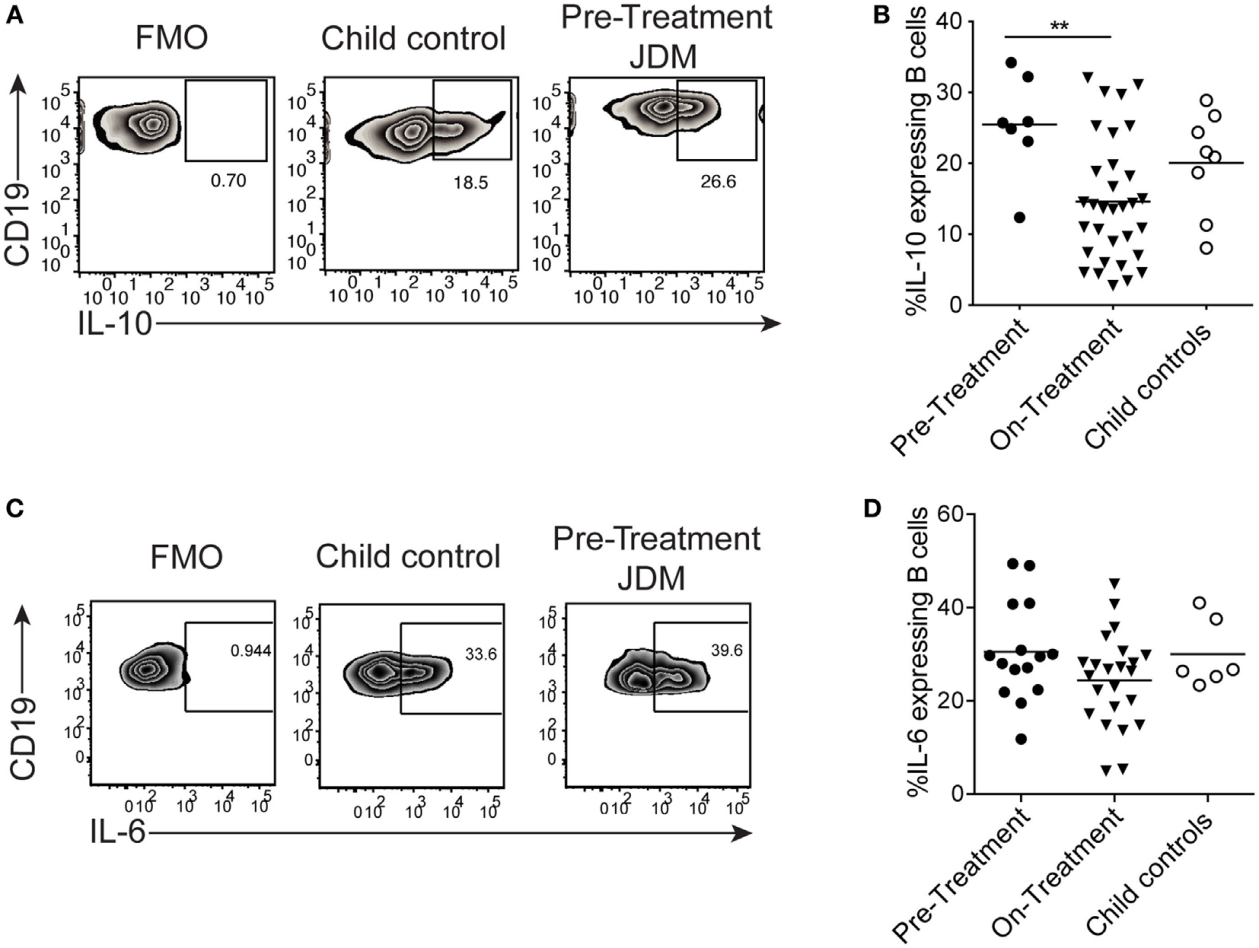
B cells from juvenile dermatomyositis (JDM) patients are able to express IL-10 upon CD40 stimulation. Peripheral blood mononuclear cells (PBMC) were stimulated with CD40L-expressing Chinese Hamster Ovary cells for 72 h, and B cell IL-10 expression was analyzed by flow cytometry, following addition of PMA, Ionomycin, and Brefeldin A for the last 4 h of culture. **(A)** Representative flow cytometry plots of B cell IL-10 expression are shown for JDM pre-treatment, on-treatment, and child controls. **(B)** The frequency of IL-10-expressing B cells after 72 h culture with CD40L stimulation is summarized for each of the groups. PBMC were stimulated for 4 h with PMA/Ionomycin **(C)** Representative flow cytometry plots of total B cell IL-6 staining are shown (left–right: FMO, child control, and JDM pre-treatment). **(D)** IL-6 expression by B cells are summarized for JDM subgroups and child controls. For panels **(B,D)**, lines represent mean values (*p < 0.05 and **p < 0.01).

We next assessed if the reduction in B cell IL-10 was an intrinsic defect in JDM patients or a signal-specific phenomenon. CD40:CD40L interactions are known to induce IL-10 and IL-6 in B cells (25). Moreover, in SLE (an IFN-driven disease), B cell IL-10 production is significantly decreased after activation with CD40L. Using a well-established co-culture assay, CD40L-expressing CHO cells were co-cultured with PBMC, and B cell IL-10 expression was analyzed after 72 h (25). We observed no significant difference in IL-10 expression between pre-treatment JDM patients and CHC. However, IL-10^+^ cells were significantly decreased in patients within the first 6 months of treatment compared with pre-treatment patients (Figures 5A,B). Immature transitional B cells from on-treatment patients were the main source of IL-10 and IL-6 upon stimulation with CD40L (Figures S6A,B in Supplementary Material).

Activation of PBMC with CD40L showed no difference in B cell IL-6 between patients and controls (data not shown). To explore this further, we examined the early kinetics of IL-6 production using PMA and Ionomycin as a stimulus. Following PMA and Ionomycin stimulation of whole PBMC, the frequency of IL-6-expressing B cells did not differ between JDM patients and controls (Figures 5C,D), and intracellular IL-10 was undetected (Figure S6C in Supplementary Material). These data suggest that the CD40 pathway, at least as interrogated by this assay, is unaffected in JDM patient B cells. These findings clearly demonstrate that B cell cytokine production is dysregulated in JDM, after activation with TLR7 and IFNα, but not with CD40L.

## Discussion

It is well established that B cells are expanded in PB of JDM (45, 57) and are thought to have an important role in JDM pathology, *via* the production of MSAs. However, to date, little is known regarding B cell phenotype and function in the context of JDM. In this study, we show that there is an expansion of immature transitional B cells in JDM patients pre-treatment and that the frequency of this population correlates with disease severity. Our data reveal that the B cell compartment in JDM exhibits a strong interferon gene signature and that this signature is associated with an increase in immature transitional B cells. Furthermore, in addition, we show that B cells from JDM patients are defective in their ability to produce IL-10, after activation with TRL7/8 agonists and IFNα.

Studies examining B cell phenotype in autoimmune diseases such as systemic sclerosis (29) and neuromyelitis optica (58) report a reduction in the number of immature transitional B cells in patients. Recently, in the context of DM, Li et al. showed that immature transitional B cells are decreased in adult DM (59). However, in this study, we report a significantly expanded immature transitional B cell population, specifically in pre-treatment JDM patients. To the best of our knowledge, this is the first report demonstrating an expansion of these cells in JDM. The mainstay of treatment for JDM patients is oral prednisolone and methotrexate, but other DMARDS and biologics were used including cyclophosphamide and azathioprine. Studies of the use of prednisolone in healthy adults and in SLE patients have shown that the frequency of B cells reduces in a time- and dose-dependant manner (60, 61). A more recent study showed that both total B cells and the immature transitional B cell subset were reduced in frequency in JIA patients treated with methotrexate (62). Equally, cyclophosphamide has also been shown to cause a general lymphocytopenia and in SLE reduce markers of B cells activation (63). We identified a significant reduction in the immature transitional B cell population across treatments and it is possible that the effect of treatment could be a confounding variable and therefore a limitation of our study.

Immature transitional B cell frequencies in the periphery decrease with age, with the frequency being highest in infancy (47). Our cohort of CHC further confirms these findings. However, immature transitional B cell frequency did not correlate with age in active JDM patients pre-treatment suggesting that normal B cell development is disturbed in patients following the onset of autoimmunity. Interestingly, immature transitional B cell frequency directly correlated with disease activity, suggesting that these cells were linked to the development of disease. Furthermore, we show that these cells in JDM patients were proliferating and have undergone more cell divisions *in vivo* than age-matched CHC, indicated by their lower KREC content. While these results indicate the replication history of B cells (48), future work should aim to distinguish whether there is a higher output of immature transitional B cells from the bone marrow and to determine the site of proliferation.

It has been previously reported that TLR-9 activated pDC, which produce IFNα, expand CD19^+^CD24^hi^CD38^hi^ B cells *in vitro* (32). Interestingly, RNAseq analysis comparing gene expression between pre-, on-treatment patients and CHC, identified a strong IFNα signature, with molecules downstream of IFNα receptor signaling (64) being highly expressed in pre-treatment patient B cells vs controls. These data suggest that the interferonopathy could be driving the expansion of immature transitional B cells in JDM *in vivo*. Indeed, we found that there was a strong positive correlation between interferon-driven chemokines and the frequency of immature transitional B cells in JDM patients. Previous studies have also identified a strong IFNα signature in muscle (10, 65), skin (66), PBMC and serum of DM and JDM patients (8, 9); a feature shared with other autoimmune diseases (67). Furthermore, pDC, which are the major producers of IFNα, are known to be abundant in skin (66, 68, 69) and muscle (7, 69) in DM and JDM patients where they could potentially crosstalk with B cells that have also infiltrated inflamed muscle tissue (69). More recently, the role of IFNβ has been suggested to be pivotal to the pathogenesis of adult DM. Elevated serum levels of IFNβ were associated with an elevated type I interferon gene signature and, moreover, correlated with skin disease activity in adult DM (70). However, the role of IFNβ in JDM disease pathology remains less well characterized. In future work, it would be important to dissect the relative contributions of IFNα and IFNβ in JDM disease pathogenesis and how this affects B cell function. Notably, clinical trials testing anti-IFNα biologics in the treatment of rheumatological disease have started to gain traction (71). Sifalimumab, a human IgG1κ monoclonal antibody, has been trialed in a phase 1b study in adult DM patients (72) and, successfully in phase IIb trial in SLE (73). Our study provides further evidence that anti-IFNα biologics could be an important treatment target in JDM.

Of particular interest, gene expression of *TLR7* and its associated downstream signaling molecule *IRF7* were significantly higher in pre- vs on-treatment B cells. It has been previously reported that activation of cells with IFNα induces IRF7 expression in multiple cell types (74) and can directly induce TLR7 expression in naïve B cells. IFNα is also required for TLR7 mediated polyclonal B cell expansion (15). Importantly, overexpression of TLR7 has been shown to increase susceptibility to autoimmunity (75) and has a role in disease pathogenesis in mouse models of lupus (76). Furthermore, overexpression of TLR7 in murine B cells promotes expansion and autoantibody production in the transitional-1 population (77). In our study, we found that while TLR7 and IFNα stimulation showed a trend to increased frequency of CD19^+^CD24^hi^CD38^hi^ B cells in controls, this was not observed in patients, possibly due to chronic activation of this pathway *in vivo*. Furthermore, we found TLR7 ligation in healthy B cells induces IL-6 and IL-10 production in child controls similarly to previous studies using B cells isolated from healthy adults (78). However, in JDM patients stimulation with the TLR7 agonist R848 favored IL-6 production over IL-10. Notably, IL-6 has been linked to disease activity in JDM (9) and is detectable in serum during active disease. Given the pre-disposition of autoantibody production in JDM (17) and that IL-6 is an important growth factor for plasmablast generation (79), it is possible that immature transitional B cells receive the required signals to differentiate to plasmablasts in muscle tissue (32).

We next investigated whether there was an intrinsic IL-10 defect in patient B cells using the CD40–CD40L interaction which is key for B cell development and induces IL-10 in B cells (80) Contrary to Kalampokis et al., who reported a reduction in Breg IL-10 in JDM patients (81), we did not observe a difference in JDM patients pre-treatment and controls with the CD40L stimulus, indicating that patient B cells can produce IL-10 with CD40 activation. These results suggest that activation through CD40 is not defective in these patients. However, the cohort in Kalampokis et al. contained patients already on-treatment and different stimuli (CD40L + CpG-B) were used, which could account for the differences seen between the studies. We show that patients on-treatment with prednisolone and methotrexate have reduced IL-10 production and immature transitional B cell frequency; and that these are proportional to each other. The increase in IL-10 seen after CD40 activation in pre-treatment JDM patients could be linked to the higher frequency of immature transitional B cells found in these patients compared with child controls. This phenomenon could also explain the reduction in B cell IL-10 in patients on-treatment, due to the decrease in immature transitional B cells with treatment. Furthermore, the decrease in immature transitional B cells itself is likely due to the effects of prednisolone and cyclophosphamide on B cell proliferation, since it is well known that glucocorticoids cause B cell lymphopenia (60). We propose that the immature transitional B cell population is capable of producing IL-10, given the right signals *in vivo*, but are skewed away from Breg differentiation and toward a pro-inflammatory phenotype by over-activation of B cells with IFNα and TLR7 agonists during disease. In support of this hypothesis, it has been reported that TLR7 and CD40 signals synergize to promote IL-6 expression in B cells (82).

In conclusion, we show that immature transitional B cells are greatly expanded in JDM blood, before treatment, correlate with disease activity, and that their frequency decreases upon treatment with prednisolone and methotrexate. This is likely caused by a reduction in inflammation, which drives the expansion of immature transitional B cells and a reduction in the proliferation of B cells by glucocorticoids. Notably, immature transitional B cells from patients re-populate to equivalent levels seen in CHC after >6 months on-treatment. We propose a model whereby TLR7 ligation and stimulation *via* IFNα lead to the expansion of immature transitional B cells, and prime them toward a pro-inflammatory state in JDM. These data support a growing body of evidence that targeting the TLR7 and IFNα response in autoimmune disease may be novel therapeutic pathways to target in DM.

## Ethics Statement

Parents and patients gave written informed consent or age-appropriate assent. The study obtained ethical approval through London-Bloomsbury and North-East Yorkshire Research Ethics Committees (MREC—01/3/022) for JDM sample collection. Child healthy control samples were donated under the North Harrow ethics committee approval (REC 11/0101). All consent was obtained in accordance with the Declaration of Helsinki.

## Author Contributions

CP, MW, CTD, GO, SD, CLD, SA, EM, and AR conducted experiments and analyzed data. CP, MW, ER, RC, YI, PB, DK, DI, CM, KN, and LW designed the study. All the authors participated in writing the manuscript and gave approval before submission.

## Conflict of Interest Statement

The authors declare that the research was conducted in the absence of any commercial or financial relationships that could be construed as a potential conflict of interest.
