# Supplementary material for: CD19+CD24hiCD38hi B Cells Are Expanded in Juvenile Dermatomyositis and Exhibit a Pro-Inflammatory Phenotype After Activation Through Toll-Like Receptor 7 and Interferon-α
Source: Front Immunol. 2018 Jun 22;9:1372. doi: 10.3389/fimmu.2018.01372 (PMC6024011; doi:10.3389/fimmu.2018.01372)
Supplement: Supplementary file 1 [file table_1.PDF]

Supplementary Tables 1-3

$CD19^{+}CD24^{hi}CD38^{hi}$ B cells are expanded in juvenile dermatomyositis and exhibit a pro-inflammatory phenotype after activation through toll-like receptor 7 and interferon-α

^Christopher JM Piper,[1] ^Meredyth GLl Wilkinson,[1,7] Claire T Deakin,[2] Georg W Otto,[3,4] Stefanie Dowle,[3,4] Chantal L Duurland,[2] Stuart Adams,[5] Emiliano Marasco,[6] Elizabeth C Rosser,[2] Anna Radziszewska,[1,7] Rita Carsetti,[6] Yiannis Ioannou,[1,7] Phil Beales,[3,4] Daniel Kelberman,[3,4] David A Isenberg,[1,7] Claudia Mauri,[1] ±Kiran Nistala,[1] ±*Lucy R Wedderburn[2,4,7] on behalf of the Juvenile Dermatomyositis Research Group.

***Correspondence:** Professor Lucy R Wedderburn: l.wedderburn@ucl.ac.uk

1 Supplementary Tables

Supplementary Table 1: 20 most significantly expressed genes by adjusted p value in a comparison of B cells from pre-and on-treatment JDM patients. A positive log fold change signifies up-regulation and negative a down-regulation of gene expression in pre-compared to on-treatment JDM.

hgnc_symbol	Description	Log fold change	p adjusted value
CMPK2	cytidine/uridine monophosphate kinase 2 [Source:HGNC Symbol;Acc:HGNC:27015]	5.015845164	1.17E-24
EIF2AK2	eukaryotic translation initiation factor 2 alpha kinase 2 [Source:HGNC Symbol;Acc:HGNC:9437]	2.204637499	5.82E-19
RSAD2	radical S-adenosyl methionine domain containing 2 [Source:HGNC Symbol;Acc:HGNC:30908]	4.495877258	7.51E-19
HERC6	HECT and RLD domain containing E3 ubiquitin protein ligase family member 6 [Source:HGNC Symbol;Acc:HGNC:26072]	2.999681795	1.94E-18
OASL	2'-5'-oligoadenylate synthetase like [Source:HGNC Symbol;Acc:HGNC:8090]	5.175624818	3.63E-17
IFI44	interferon induced protein 44 [Source:HGNC Symbol;Acc:HGNC:16938]	3.619703923	6.44E-17
OAS3	2'-5'-oligoadenylate synthetase 3 [Source:HGNC Symbol;Acc:HGNC:8088]	3.759215718	6.44E-17
IFIT1	interferon induced protein with tetratricopeptide repeats 1 [Source:HGNC Symbol;Acc:HGNC:5407]	5.661457062	2.58E-16
IFITM1	interferon induced transmembrane protein 1 [Source:HGNC Symbol;Acc:HGNC:5412]	2.65927262	3.71E-16
MX2	MX dynamin like GTPase 2 [Source:HGNC Symbol;Acc:HGNC:7533]	2.075976163	5.91E-16
USP18	ubiquitin specific peptidase 18 [Source:HGNC Symbol;Acc:HGNC:12616]	4.478369432	1.71E-15
DDX60	DExD/H-box helicase 60 [Source:HGNC Symbol;Acc:HGNC:25942]	2.502981805	3.81E-15
IFI44L	interferon induced protein 44 like [Source:HGNC Symbol;Acc:HGNC:17817]	4.3932757	4.30E-15
NRIR	negative regulator of interferon response (non-protein coding) [Source:HGNC Symbol;Acc:HGNC:51269]	4.367969389	7.08E-15
HERC5	HECT and RLD domain containing E3 ubiquitin protein ligase 5 [Source:HGNC Symbol;Acc:HGNC:24368]	3.122510334	2.81E-14
IFIT3	interferon induced protein with tetratricopeptide repeats 3 [Source:HGNC Symbol;Acc:HGNC:5411]	5.203493029	3.65E-14
PLSCR1	phospholipid scramblase 1 [Source:HGNC Symbol;Acc:HGNC:9092]	2.422234425	4.44E-14
IFI6	interferon alpha inducible protein 6 [Source:HGNC Symbol;Acc:HGNC:4054]	3.059370394	1.51E-13
SLFN5	schlafen family member 5 [Source:HGNC Symbol;Acc:HGNC:28286]	2.935522167	1.81E-13
STAT1	signal transducer and activator of transcription 1 [Source:HGNC Symbol;Acc:HGNC:11362]	2.412777888	1.13E-12

Supplementary Table 2: 20 most significantly expressed genes by adjusted p value in a comparison of B cells from pre-treatment JDM patients and child healthy controls. A positive log fold change signifies up-regulation and negative a down-regulation of gene expression in pre-treatment JDM compared to child healthy control.

**hgnc_symbol**	**Description**	**Log fold change**	**p adjusted value**
CMPK2	cytidine/uridine monophosphate kinase 2 [Source:HGNC Symbol;Acc:HGNC:27015]	4.125949196	2.77E-07
EIF2AK2	eukaryotic translation initiation factor 2 alpha kinase 2 [Source:HGNC Symbol;Acc:HGNC:9437]	2.022581215	2.77E-07
OASL	2'-5'-oligoadenylate synthetase like [Source:HGNC Symbol;Acc:HGNC:8090]	5.097639577	8.76E-07
ADAR	adenosine deaminase, RNA specific [Source:HGNC Symbol;Acc:HGNC:225]	1.079673516	1.99E-06
TGFBI	transforming growth factor beta induced [Source:HGNC Symbol;Acc:HGNC:11771]	-2.889143829	2.50E-06
IFITM1	interferon induced transmembrane protein 1 [Source:HGNC Symbol;Acc:HGNC:5412]	2.429796925	5.49E-06
MX2	MX dynamin like GTPase 2 [Source:HGNC Symbol;Acc:HGNC:7533]	1.875673164	5.49E-06
NRIR	negative regulator of interferon response (non-protein coding) [Source:HGNC Symbol;Acc:HGNC:51269]	4.217600512	8.35E-06
CD4	CD4 molecule [Source:HGNC Symbol;Acc:HGNC:1678]	-2.522215473	8.35E-06
HERC5	HECT and RLD domain containing E3 ubiquitin protein ligase 5 [Source:HGNC Symbol;Acc:HGNC:24368]	2.967715524	1.16E-05
HERC6	HECT and RLD domain containing E3 ubiquitin protein ligase family member 6 [Source:HGNC Symbol;Acc:HGNC:26072]	2.434812047	1.21E-05
BAHCC1	BAH domain and coiled-coil containing 1 [Source:HGNC Symbol;Acc:HGNC:29279]	3.539876725	1.28E-05
SLFN5	schlafen family member 5 [Source:HGNC Symbol;Acc:HGNC:28286]	2.861670142	1.29E-05
LGALS3BP	galectin 3 binding protein [Source:HGNC Symbol;Acc:HGNC:6564]	2.671699546	1.29E-05
IL2RA	interleukin 2 receptor subunit alpha [Source:HGNC Symbol;Acc:HGNC:6008]	-2.202209619	1.52E-05
OAS3	2'-5'-oligoadenylate synthetase 3 [Source:HGNC Symbol;Acc:HGNC:8088]	3.143704429	1.81E-05
IFI44	interferon induced protein 44 [Source:HGNC Symbol;Acc:HGNC:16938]	3.00981476	1.96E-05
STAT1	signal transducer and activator of transcription 1 [Source:HGNC Symbol;Acc:HGNC:11362]	2.361882153	1.96E-05
DDX60	DExD/H-box helicase 60 [Source:HGNC Symbol;Acc:HGNC:25942]	2.179692129	1.96E-05
MDFIC	MyoD family inhibitor domain containing [Source:HGNC Symbol;Acc:HGNC:28870]	-1.257982447	3.17E-05

Supplementary Table 3: 20 most significantly expressed genes by adjusted p value in a comparison of B cells from on-treatment JDM patients and child healthy controls. A positive log fold change signifies up-regulation and negative a down-regulation of gene expression in on-treatment JDM compared to child healthy control.

hgnc_symbol	Description	Log fold change	p adjusted value
CD2	CD2 molecule [Source:HGNC Symbol;Acc:HGNC:1639]	-4.027058769	3.71E-06
FYB	FYN binding protein [Source:HGNC Symbol;Acc:HGNC:4036]	-1.736065003	0.000654737
CD3D	CD3d molecule [Source:HGNC Symbol;Acc:HGNC:1673]	-3.387862675	0.000654737
UTS2	urotensin 2 [Source:HGNC Symbol;Acc:HGNC:12636]	-5.792144829	0.001298924
CD3E	CD3e molecule [Source:HGNC Symbol;Acc:HGNC:1674]	-2.818016494	0.001298924
BRD7	bromodomain containing 7 [Source:HGNC Symbol;Acc:HGNC:14310]	-0.753749825	0.001298924
SESN1	sestrin 1 [Source:HGNC Symbol;Acc:HGNC:21595]	-1.695533365	0.002503248
CD4	CD4 molecule [Source:HGNC Symbol;Acc:HGNC:1678]	-2.07007882	0.002503248
GJB6	gap junction protein beta 6 [Source:HGNC Symbol;Acc:HGNC:4288]	-5.515063758	0.003423732
GPA33	glycoprotein A33 [Source:HGNC Symbol;Acc:HGNC:4445]	-1.534820483	0.004655566
IL7R	interleukin 7 receptor [Source:HGNC Symbol;Acc:HGNC:6024]	-2.668892123	0.006919731
Unknown	Unknown	-1.737965221	0.006919731
UNC50	unc-50 inner nuclear membrane RNA binding protein [Source:HGNC Symbol;Acc:HGNC:16046]	-0.632431247	0.014092006
XIST	X inactive specific transcript (non-protein coding) [Source:HGNC Symbol;Acc:HGNC:12810]	-3.081795412	0.014092006
ANXA1	annexin A1 [Source:HGNC Symbol;Acc:HGNC:533]	-2.136255667	0.014092006
EDAR	ectodysplasin A receptor [Source:HGNC Symbol;Acc:HGNC:2895]	-4.629933298	0.024296951
CRCP	CGRP receptor component [Source:HGNC Symbol;Acc:HGNC:17888]	0.684787954	0.024296951
CCDC6	coiled-coil domain containing 6 [Source:HGNC Symbol;Acc:HGNC:18782]	-0.58270338	0.024296951
TGFBI	transforming growth factor beta induced [Source:HGNC Symbol;Acc:HGNC:11771]	-1.890063253	0.032839454
CD247	CD247 molecule [Source:HGNC Symbol;Acc:HGNC:1677]	-1.466135268	0.035328358
