# Supplementary material for: CD19+CD24hiCD38hi B Cells Are Expanded in Juvenile Dermatomyositis and Exhibit a Pro-Inflammatory Phenotype After Activation Through Toll-Like Receptor 7 and Interferon-α
Source: Front Immunol. 2018 Jun 22;9:1372. doi: 10.3389/fimmu.2018.01372 (PMC6024011; doi:10.3389/fimmu.2018.01372)
Supplement: Supplementary file 2 [file data_sheet_1.PDF]

Supplementary Figures 1-6

CD19$^+$CD24^{hi}CD38^{hi} B cells are expanded in juvenile dermatomyositis and exhibit a pro-inflammatory phenotype after activation through toll-like receptor 7 and interferon-α

^Christopher JM Piper,[1] ^Meredyth GLl Wilkinson,[1,7] Claire T Deakin,[2] Georg W Otto,[3,4] Stefanie Dowle,[3,4] Chantal L Duurland,[2] Stuart Adams,[5] Emiliano Marasco,[6] Elizabeth C Rosser,[2] Anna Radziszewska,[1,7] Rita Carsetti,[6] Yiannis Ioannou,[1,7] Phil Beales,[3,4] Daniel Kelberman,[3,4] David A Isenberg,[1,7] Claudia Mauri,[1] ±Kiran Nistala,[1] ±*Lucy R Wedderburn[2,4,7] on behalf of the Juvenile Dermatomyositis Research Group.

***Correspondence:** Professor Lucy R Wedderburn: l.wedderburn@ucl.ac.uk

1 Supplementary Figures

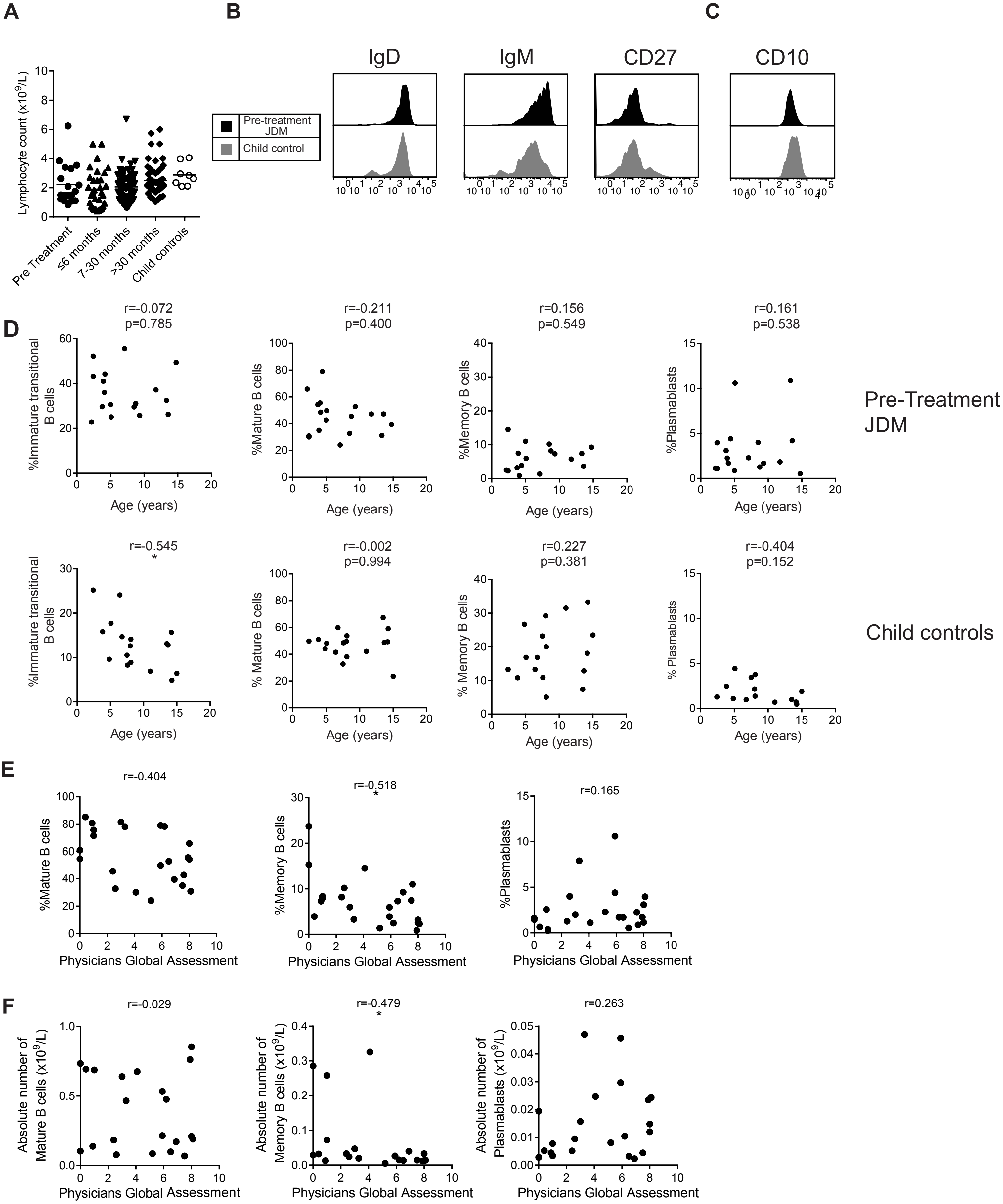

Figure S1: B cell subset frequencies correlated with age and disease activity. (A) Lymphocyte counts x 10^9 per litre of blood, for pre-treatment and on-treatment JDM patients and age matched child controls. Lines represent mean values. (B) Representative histograms of IgD, IgM and CD27 (left to right) and (C) CD10 expression gated on immature transitional B cells are shown for pre-treatment JDM patients (black outline) and child controls (grey outline). (D) Frequencies of immature transitional, mature, memory B cells and plasmablasts (left to right) analysed for correlation with age in pre-treatment patients (top row) and child controls (bottom row). For patients up to 6 months into treatment (including pre-treatment), the (E) frequency and (F) absolute number of cells were correlated to PGA for mature, memory and plasmablasts (left to right). For figure A, lines represent mean values. Pearson r values are shown for figure D; Spearman r values for figure E. *$p<0.05$

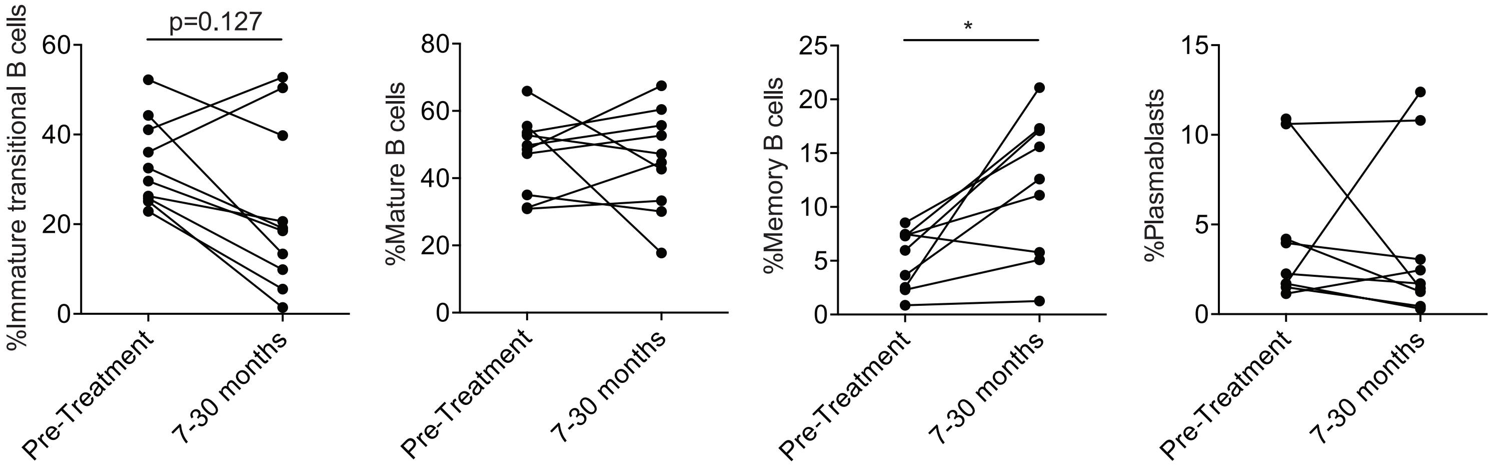

Figure S2: B cell subset frequencies in paired pre-treatment and on-treatment JDM patients. Frequencies of immature transitional B cells, mature B cells, memory B cells and plasmablasts (left-right) in patients with serial samples collected pre- and on-treatment (7-30 months). *p<0.05

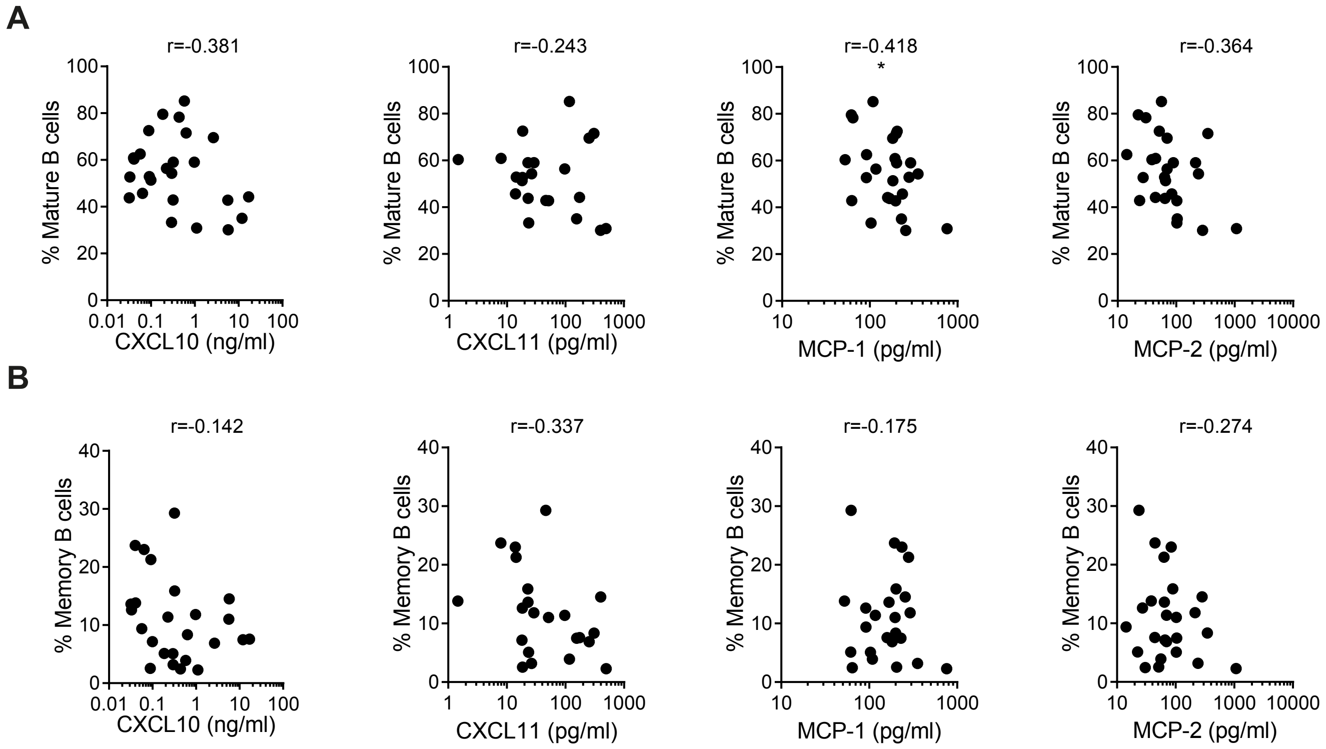

Figure S3: Correlation between type 1 interferon signature chemokines and mature and memory B cell frequencies in JDM patients. Patient sera and PBMC collected on the same day were used to assess correlations between (A) Mature and (B) Memory B cell frequency and serum concentrations of CXCL10, CXCL11, MCP-1 and MCP-2 (left to right) for all JDM patients (pre/on-treatment). *p<0.05

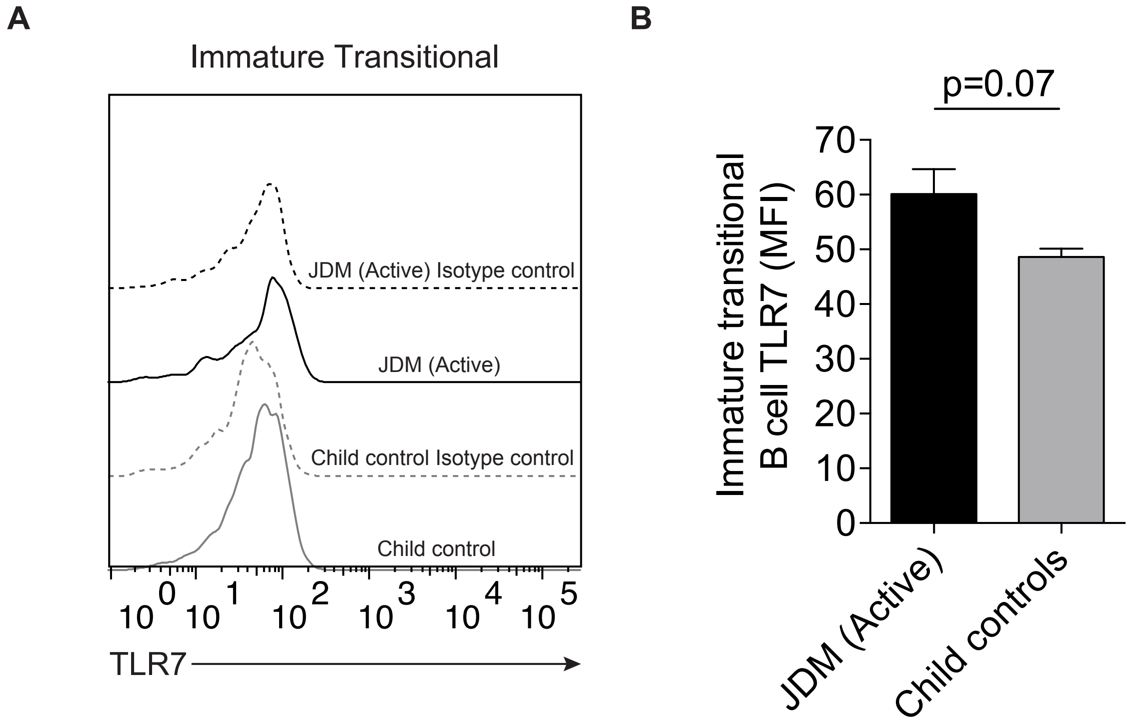

Figure S4: Immature transitional B cells from JDM patients express higher levels of TLR7 than child controls. JDM (active) patients and child control PBMC were stained intracellularly for TRL7. (A) A representative histogram of TLR7 staining in immature transitional B cells are shown for patients and controls. (B) Bar graph summarising immature transitional B cell TLR7 MFI for patients and controls (N=3 for each group). For figure B, bars represent mean ± SEM.

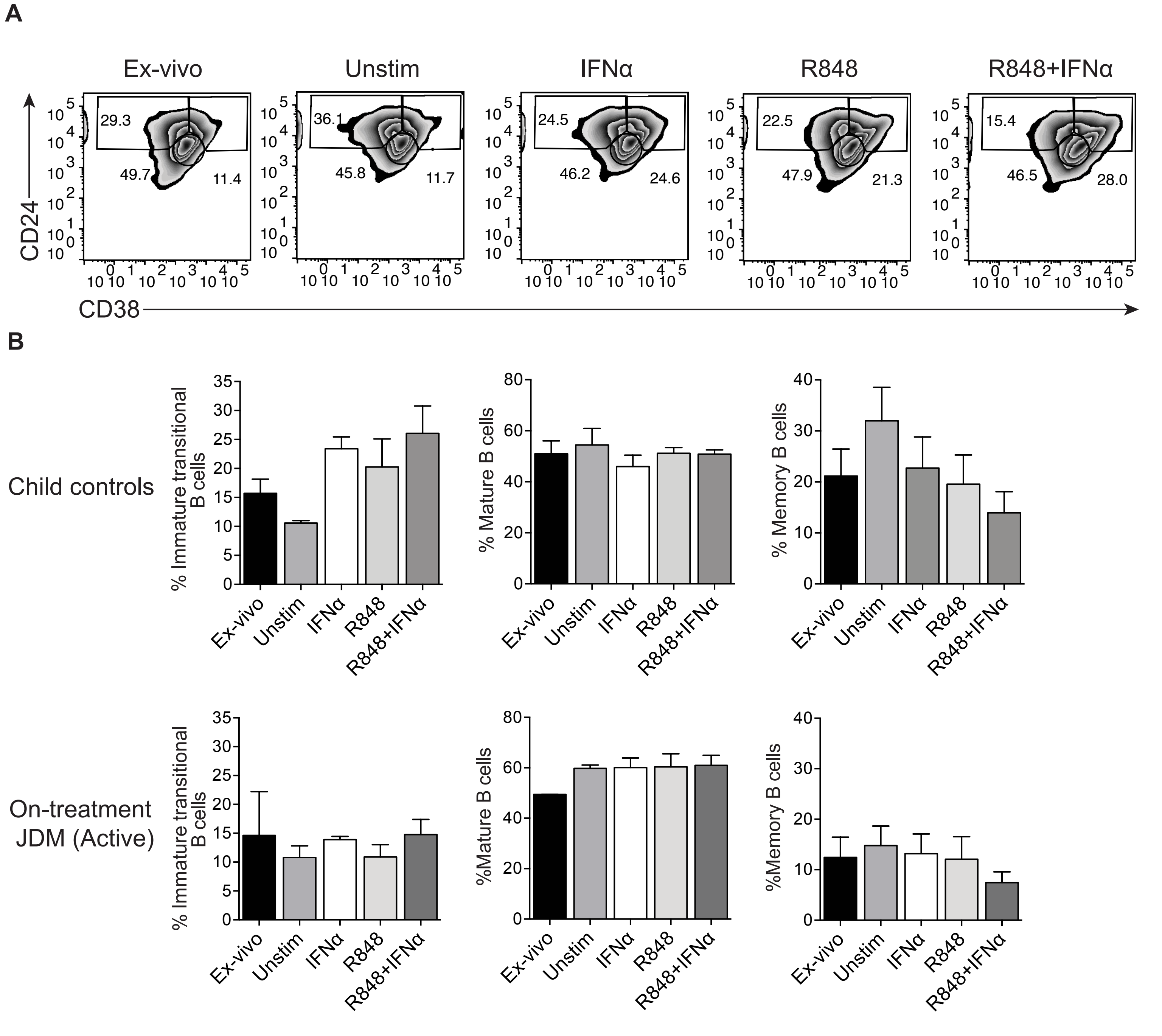

Figure S5. Child control and JDM B cell subset frequencies after culture with R848 and IFNα. B cells purified from active on-treatment patients (patients with flares had high disease activity (PGA >3.5) and were >6months into treatment), and age-matched child control PBMC, were stimulated with IFNα (1000 IU/ml), 1μg/ml R848 (a TLR7/8 agonist) or both, for 48h. B cells were stained for CD24 and CD38 expression. (A) Representative flow cytometry plots showing B cells subset gating for each of the conditions are shown (left to right: ex-vivo, unstimulated, IFNα, R848, R848+IFNα) using a representative child control sample. (B) Percentage of immature transitional, mature and memory B cells (left-right) are summarised for each culture condition for child controls (top row) and JDM patients on-treatment (bottom row). For figure B, bars represent mean ± SEM.

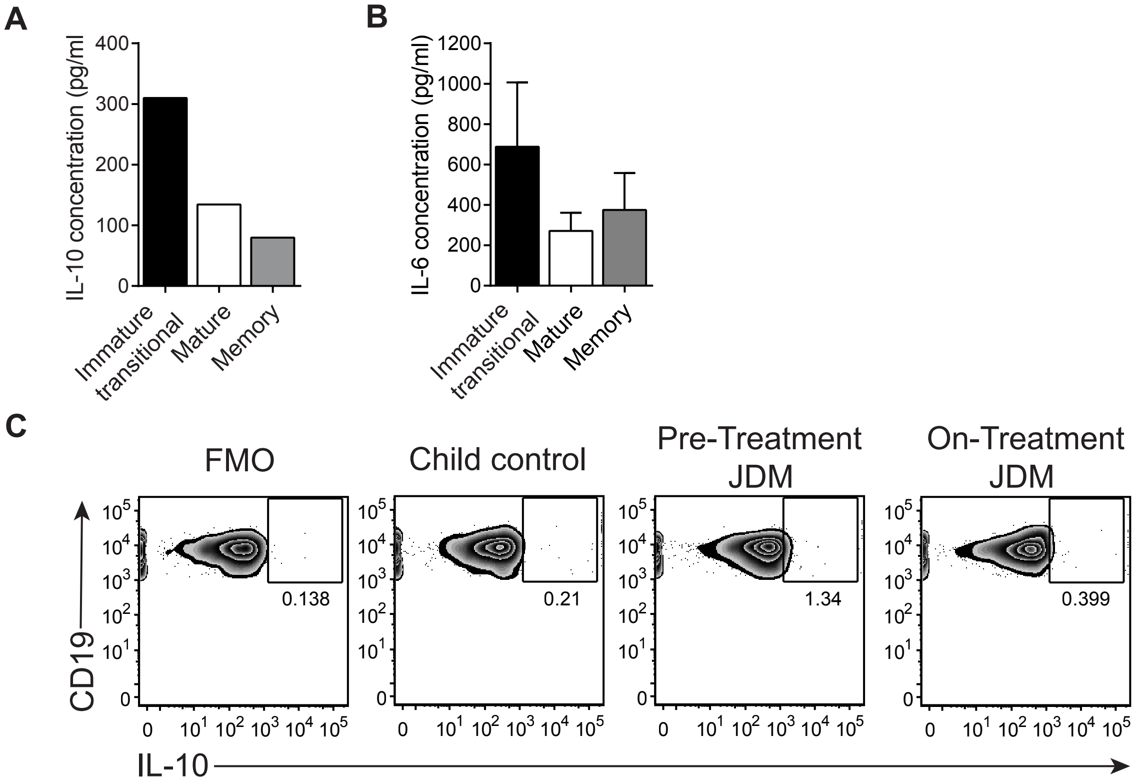

Figure S6: IL-10 and IL-6 concentrations from sorted B cell subsets from JDM patients after stimulation with CD40L. Purified B cells subsets from on-treatment JDM patients were sorted and stimulated with CD40L CHO for 72h and concentrations of (A) IL-10 and (B) IL-6 were quantified from the cell culture supernatants (N of 1 and 2 respectively). Bars represent mean +SEM. (C) PBMC were stimulated for 4h with PMA and Ionomycin in the presence of Golgi plug and the percentage of IL-10+ B cells were assessed by flow cytometry. Representative flow cytometry plots showing the percentage of IL-10+ B cells are shown for child controls and JDM patients pre- and on-treatment. FMO plot also shown. For figure B, bars represent mean ± SEM.
