# Supplementary material for: CD19+CD24hiCD38hi B Cells Are Expanded in Juvenile Dermatomyositis and Exhibit a Pro-Inflammatory Phenotype After Activation Through Toll-Like Receptor 7 and Interferon-α
Source: Front Immunol. 2018 Jun 22;9:1372. doi: 10.3389/fimmu.2018.01372 (PMC6024011; doi:10.3389/fimmu.2018.01372)
Supplement: Supplementary file 3 [file data_sheet_2.PDF]

Supplementary Methods

$CD19^{+}CD24^{hi}CD38^{hi}$ B cells are expanded in juvenile dermatomyositis and exhibit a pro-inflammatory phenotype after activation through toll-like receptor 7 and interferon-α

^Christopher JM Piper,[1] ^Meredyth GLl Wilkinson,[1,7] Claire T Deakin,[2] Georg W Otto,[3,4] Stefanie Dowle,[3,4] Chantal L Duurland,[2] Stuart Adams,[5] Emiliano Marasco,[6] Elizabeth C Rosser,[2] Anna Radziszewska,[1,7] Rita Carsetti,[6] Yiannis Ioannou,[1,7] Phil Beales,[3,4] Daniel Kelberman,[3,4] David A Isenberg,[1,7] Claudia Mauri,[1] ±Kiran Nistala,[1] ±*Lucy R Wedderburn[2,4,7] on behalf of the Juvenile Dermatomyositis Research Group.

***Correspondence:** Professor Lucy R Wedderburn: l.wedderburn@ucl.ac.uk

1 Supplementary Methods

1.1 Library preparation

Samples were processed using Illumina's TruSeq Stranded mRNA LT sample preparation kit (p/n RS-122-2101) according to manufacturer's instructions with minor alterations. Briefly, mRNA was isolated from 250 ng total RNA using Oligo dT beads to pull down Poly-Adenylated transcripts. The purified mRNA was fragmented using chemical fragmentation (heat and divalent metal cation) for 10 minutes and primed with random hexamers. Strand-specific first strand cDNA was generated using Reverse Transcriptase and Actinomycin D. This allows for RNA dependent synthesis while preventing spurious DNA-dependent synthesis. The second cDNA strand was synthesised using dUTP in place of dTTP, to mark the second strand. The resultant cDNA is then "A-tailed" at the 3' end to prevent self-ligation and adapter dimerisation. Full length TruSeq adaptors, containing a T overhang are ligated to the A-Tailed cDNA. These adaptors contain sequences that allow the libraries to be uniquely identified by way of a 6 bp Index sequence. Successfully ligated fragments were enriched with 14 cycles of PCR. The polymerase is unable to read through uracil, so only the first strand is amplified, thus making the library strand specific.

1.2 Sequencing

Libraries to be multiplexed in the same run were pooled in equimolar quantities, calculated from Qubit and Bioanalyser fragment analysis. Samples were sequenced on the NextSeq 500 instrument (Illumina, San Diego, US) using a 43 bp paired end run resulting in >15million reads per sample.

1.3 iData Analysis

Run data were demultiplexed and converted to fastq files using Illumina's bcl2fastq Conversion Software v2.16. Sequencing reads (in fastq format) were aligned to the GRCh38 reference sequence using TopHat v2.1.0.[1] Alignments were processed using samtools version 1.2 and Picard tools version 1.140 (http://picard.sourceforge.net/).[2] Aligned reads were filtered for mapq >= 4, i.e. uniquely mapping reads, and putative PCR duplicates were removed. Read summarization was performed using featureCounts.[3] Expression analysis was carried out using R version 3.2.2 [4], and differential gene expression was analysed using edgeR.[5]

1.4 GSEA

GO term and pathway enrichment analysis was carried out using '*goseq*' which uses a test based on the Wallenius' noncentral hypergometric distribution.[6] Gene set enrichment analysis was carried out with "Hallmark" gene sets from databases.[7] Normalized enrichment scores (NES) and multiple adjusted p-values (q values) were calculated.

Supplementary references

1. Kim D, Pertea G, Trapnell C, Pimentel H, Kelley R, Salzberg SL. TopHat2: accurate alignment of transcriptomes in the presence of insertions, deletions and gene fusions. *Genome Biol* 2013;14(4):R36. doi: 10.1186/gb-2013-14-4-r36
2. Li H, Handsaker B, Wysoker A, Fennell T, Ruan J, Homer N, et al. The Sequence Alignment/Map format and SAMtools. *Bioinformatics* 2009;25(16):2078-9. doi: 10.1093/bioinformatics/btp352
3. Liao Y, Smyth GK, Shi W. featureCounts: an efficient general purpose program for assigning sequence reads to genomic features. *Bioinformatics* 2014;30(7):923-30. doi: 10.1093/bioinformatics/btt656
4. Team RC. R: A Language and Enviroment for Statistical Computing. *R Foundation for Statistical Computing* 2015
5. Robinson MD, McCarthy DJ, Smyth GK. edgeR: a Bioconductor package for differential expression analysis of digital gene expression data. *Bioinformatics* 2010;26(1):139-40. doi: 10.1093/bioinformatics/btp616
6. Young MD, Wakefield MJ, Smyth GK, Oshlack A. Gene ontology analysis for RNA-seq: accounting for selection bias. *Genome Biol* 2010;11(2):R14. doi: 10.1186/gb-2010-11-2-r14
7. Subramanian A, Tamayo P, Mootha VK, Mukherjee S, Ebert BL, Gillette MA, et al. Gene set enrichment analysis: a knowledge-based approach for interpreting genome-wide expression profiles. *Proc Natl Acad Sci U S A* 2005;102(43):15545-50. doi: 10.1073/pnas.0506580102
